# Oncological needs in transgender patients

**DOI:** 10.1007/s12094-025-03917-x

**Published:** 2025-04-18

**Authors:** Francisco Ayala de la Peña, David Martínez-Ramos, Oscar Juan-Vidal, Marcelino Gómez-Balaguer, Maria Miguélez, David Páez, Virginia Arrazubi, Carmen Hinojo González

**Affiliations:** 1 Department of Medical Oncology, Hospital Universitario Morales Meseguer, Murcia, Spain; 2 Department of Medicine, Universidad de Murcia, Avda. Marqués de los Vélez, s/n, 30008 Murcia, Spain; 3 Breast Unit, General and Digestive Surgery Department, Hospital General Universitari Castelló, Castelló, Spain; 4 Department of Medical Oncology, Hospital Universitari i Politècnic La Fe, Valencia, Spain; 5 Gender Identity Unit, Endocrinology and Nutrition Section, Hospital Universitario Doctor Peset-Fundación FISABIO, Valencia, Spain; 6 Gender Identity Unit, Endocrinology Service, Hospital General Universitario Gregorio Marañón, Madrid, Spain; 7 Medical Oncology Department, Hospital de la Santa Creu i Sant Pau, Barcelona, Spain; 8 Medical Oncology Department, Hospital Universitario de Navarra, Pamplona, Spain; 9 Instituto de Investigación Sanitaria de Navarra (IdiSNA), Pamplona, Spain; 10 Hospital Universitario Marqués de Valdecilla, Santander, Spain; 11 Instituto de Investigación Valdecilla (IDIVAL), Santander, Spain

**Keywords:** Breast cancer, Cancer risk, Gender-affirming care, Prostate cancer, Screening, Transgender, Spain

## Abstract

Transgender people encounter unique health disparities in oncology. They face substantial barriers in accessing healthcare that are exaggerated by bias/discrimination from healthcare professionals and systems, and socio-economic marginalisation. This review explores the current landscape of cancer risk, screening and management in transgender individuals from a Spanish perspective. Nationwide data are lacking, but estimates from Madrid suggest that 22 per 100,000 individuals are transgender. The needs of the transgender individual for gender-affirming surgeries and gender-affirming hormone therapy may alter the individual’s oncological risk profile and likelihood of receiving appropriate screening, and when diagnosed with cancer may have to be balanced against treatment requirements (e.g., endocrine therapy for breast cancer). There remain unmet needs in the oncological care of the transgender patient. Concerted effort is required to address clinical research gaps, and reform healthcare education and policy, in order to develop inclusive clinical practices that enhance patient care and outcomes.

## Introduction

The term “transgender” describes people whose gender identity differs from the sex assigned to them at birth [1]. Recent estimates suggest that approximately 0.5% of the population of the United States of America (USA) identify as transgender, but specific data for Spain remain scarce [1].

Transgender people constitute a medically underserved population that encounters unique health disparities, including in the realm of oncology. Transgender individuals face substantial barriers in accessing healthcare, which are compounded by biases of healthcare professionals (HCPs) and a general lack of knowledge about best practices in transgender health [2, 3]. These barriers are influenced by socio-economic marginalisation, lack of health insurance and discrimination within the healthcare system [4, 5].

This review aims to explore the current landscape of cancer risk, screening and management in transgender populations, with a particular focus on the gaps in knowledge and healthcare provision in Spain. It seeks to delineate the specific oncological needs of transgender patients and to highlight the necessary shifts in clinical practice and research to accommodate these needs effectively. As such, the authors of this review represent both the specialities of oncology and endocrinology.

## Methods

A systematic search of the PubMed, Google Scholar and Medline databases was conducted in April and in June 2024, using the following search terms (and synonyms of them): “transgender”, “cancer”, “gender”, “gender identity”, “assigned male at birth”, “assigned female at birth”, “gender-affirming hormone therapy”, “gender-affirming surgery”, “gender-inclusive”, “gender identity”, “gender-sensitive”, “risk factors”, “genetic variants”, “gene mutation”, “vaginoplasty”, “hysterectomy”, “neovagina”, “oophorectomy”, “orchiectomy”, “vaccinations”, “breast cancer’, “ovarian cancer”, “cervical cancer”, “endometrial cancer”, “prostate cancer”, “testicular cancer”, “anal cancer”, “rectal cancer”, “screening”, “guidelines”, “early detection”, “disparity”, “barriers”, “oncological challenges”, “access”, “outcomes”, “cancer care”, “experiences” and “Spain”. Articles identified from this search were evaluated for relevance and inclusion in this review. Additional articles were identified from the bibliographies of included articles, and from additional PubMed searches that aimed to answer specific research questions. All types of articles published in English, predominantly in the previous 10 years were considered in this review.

The following guidelines were used: NCCN, American College of Radiology (ACR), World Professional Association for Transgender Health (WPATH), American Association of Clinical Endocrinologists, American Society of Andrology, The American College of Obstetricians and Gynaecologists (ACOG), European Society for Pediatric Endocrinology, European Society of Endocrinology, Pediatric Endocrine Society and Veterans Health Administration, Canadian Breast Screening Guidelines and Guidelines and Protocols for Comprehensive Primary Care for Trans Clients [5–15].

## Definitions

Understanding the terminology related to transgender identities is crucial for HCPs to provide respectful and effective care (Table 1). “Transgender” is an umbrella term that refers to diversity in gender experience and is distinct from the term “transsexual”, which is now considered outdated [16]. An increasing number of transgender people identify themselves as non-binary [17, 18] and may request different medical and surgical treatments to those who do not identify as non-binary [19]; this may influence their oncological risk. This review does not discuss non-binary individuals.

**Table 1:** Definitions of key terms relating to transgender individuals

Term	Definition
Gender	Gender is a social, cultural and psychological construct. It refers to the social roles, attributes and opportunities associated with being born with one set of genitalia or another. These attributes are learned in society and clearly vary according to geography, culture and religion, and are modifiable with time and social progress. Gender determines what is expected, valued, allowed and admitted in a woman or a man at any given moment. From an academic point of view, we can identify three components of gender: gender identity, gender expression and biological sex
Gender identity	This conveys the personal sense of belonging to a gender. It divides people into men, women and non-binary people. It refers to the internal and individual experience of gender. The experience of gender is intimate and diverse. It answers the question, “Who am I?” Gender identity is self-determined and is not diagnosed by others
Gender expression	External or social manifestation of gender identity. It refers to the social labels attached to being male or female. Gender expression can be masculine, feminine, androgynous or variable. It can be represented by clothing, accessories, hairstyles, make-up and body language, etc. It is learned in society during upbringing. Gender identity and gender expression do not always coincide in a normative way
Biological sex	The set of biological characteristics that define all human beings as male and female and give rise to a phenotype with a male or female appearance. Intersex variants exist in very low proportions. Biological sex is determined by chromosomes, hormones and internal and external sexual organs
Sexuality	The way in which people experience and express themselves as sexed beings. It is the result of the interaction of biological, psychological, socio-economic, cultural, ethical, religious or spiritual factors
Sexual orientation	The sexual preference or inclination that characterises the object of a person’s amorous or erotic desires
Transgender	A person whose gender identity differs from that assigned at birth, based on the appearance of their external genitalia and on their educated gender
Cisgender	A person whose gender identity matches that assigned at birth
Trans	An umbrella term or expression that reflects the different sexogenic realities or sexogenic diversities, and aims to cover heterogeneity in the conception of each person’s body, identity and experiences, which go beyond learned social norms and stereotypes. It is intended to be an inclusive term and is increasingly being used in the literature. It includes people both with and without gender-affirming medical treatment
Transsexuality	A former ICD- 10 term indicating a mismatch between the biological sex defined by the genitalia assigned at birth and the person’s gender identity. It is no longer in use
Transsexual	This term was used to denote a person who feels that they were born with a physical sex not in accordance with their gender identity. It was also used to define a person who presents physical changes due to hormonal or surgical treatment or who is about to start them. It is no longer in use because it focuses on anatomical aspects
Gender identity disorder	A pathologising term that should not be used. It should not be used because it stigmatises the individual, and has been removed from all guidelines and catalogues
Gender Dysphoria	Clinically significant distress or “discomfort” caused by a mismatch between sex assigned at birth and gender felt. Gender dysphoria is recognised as a pathology in the American Psychiatric Association’s DSM- 5, although not every transgender person has dysphoria. In the current literature, the term gender dysphoria refers to a spectrum of symptoms (if present) and is not a diagnostic term
Trans woman	Female in identity. Refers to the “male to female” of Anglo-Saxon literature
Trans man	Male in identity. Refers to the “female to male” of Anglo-Saxon literature
Non-binary genders	These are different ways of feeling, living and expressing identities that go beyond stereotypes and labels. They seek to go beyond the male–female binomial
AMAB	Non-binary person assigned male at birth
AFAB	Non-binary person assigned female at birth
Transition	Process of adapting physical and/or social and/or administrative characteristics to those of their gender identity. It can also be the moment when a transgender person presents him/herself in society according to his/her gender identity. There are different transitions, different speeds of transition and different ways of experiencing it. It is a personal choice for each person to choose the type of transition
Cispassing	The desire of a transgender individual to acquire all the external somatic social characteristics of the persons with whose identity they identify, resorting to all necessary medical/surgical procedures

*AFAB* assigned female at birth, *AMAB* assigned male at birth, *DSM- 5* Diagnostic and Statistical Manual of Mental Disorders, *ICD* International Statistical Classification of Diseases and Related Health Problems

“Gender dysphoria” can influence a person’s decision to seek gender-affirming treatments [20]. Gender-affirming care (GAC) encompasses medical or surgical interventions intended to align an individual’s physical characteristics with their gender identity. This may include hormone therapy, chest/breast surgery, genital surgery and other therapies and procedures [21].

The terms “assigned male at birth” (AMAB) and “assigned female at birth” (AFAB) describe the sex assignment given at birth (typically based on external anatomy), and are increasingly used in medical contexts to avoid conflating biological and gender identity [9]. Understanding and using correct terminology is a practical necessity for ensuring that all patients receive respectful, competent and culturally sensitive medical care.

## Epidemiology of the transgender population

Transgender individuals, though a relatively small proportion of the global population, represent a significant and expanding demographic with distinct healthcare needs. In the USA, recent estimates indicate that the transgender population includes approximately 1.6 million individuals [2, 4]. Of these, over 400,000 transgender individuals in the USA are likely to be diagnosed with cancer during their lifetimes [4].

Nationwide population estimates for transgender people in Spain are not available at present. However, two Spanish studies investigated demand for transgender-specific healthcare at the gender identity unit of public hospitals [16]. The Ramón y Cajal University Hospital study estimated that the prevalence of transgender individuals in the Madrid region was 22.1 per 100,000 individuals: 31.2 per 100,000 were transgender female (AMAB) and 12.9 per 100,000 were transgender male (AFAB) [22]. The Dr Peset University Hospital study examined trends over time, observing that the annual number of referrals to one gender clinic rose from 18 in 2012 to 189 in 2021, marking a 1050% increase [16]. This surge was most pronounced among adolescents and young adults, particularly those AFAB. In contrast, the assigned sex ratio among children, adults and older adults favoured individuals AMAB [16].

These trends reflect a significant shift in the socio-demographic profile of individuals seeking gender-affirming care in Spain, indicating not only a rise in demand but also a diversification of the transgender population engaging with healthcare services.

## GAC and cancer risk

GAC encompasses a range of medical interventions aimed at aligning an individual’s physical appearance with their gender identity. It includes gender-affirming hormone therapy (GAHT) and gender-affirming surgery (GAS). It is essential for patients to engage in thorough consultations with HCPs to fully understand the implications of these treatments, particularly regarding long-term health impacts such as fertility and cancer risk.

GAHT plays a pivotal role in the physical transformation process and requires a comprehensive physical, psychological and sociological assessment prior to initiation. Feminising hormone therapy typically includes oestradiol and androgen blockers, while masculinising therapy relies on testosterone (Table 2). In addition, gestagens (progesterone and derivatives) are used in transgender men to induce amenorrhea, provide contraception and manage chronic pelvic pain. Gestagens are also used in transgender women to achieve adequate breast development, where this proves insufficient with oestrogen only. For younger transgender individuals in prepubertal stages (Tanner stages II/III), gonadotropin hormone-releasing hormone (GnRH) analogues may be used to block puberty that is incongruent with the individual’s gender identity.

**Table 2:** Most commonly used gender-affirming hormone therapy drugs in Spain [71]

Type of agent	Mechanism of action/active principle	Active drug
Antiandrogens	GnRH analogues	Triptorelin
	Progestogens	Cyproterone acetate Dienogest Drospirenone Nomegestrol acetate
	Testosterone receptor antagonists	Spironolactone
	5α-reductase inhibitors	Finasteride Dutasteride
Feminising preparations	Oestrogens	Micronised oestradiol Oestradiol valerate Oestradiol hemihydrate^a^
	Progesterone	Micronised progesterone
Masculinising preparations	Testosterone	Testosterone cypionate Testosterone undecanoate Testosterone^b^
Preparations to stop menstruation^c^	Progestogens	Norethisterone Medroxyprogesterone acetate Micronised progesterone Etonogestrel Levonorgestrel

Adapted with permission from Pérez López [71]. https://doi.org/10.1016/j.endien.2022.11.007

^a^ Available in Spain in gel, patch or spray formulations

^b^ Available in Spain in a gel formulation

^c^ Progestogens may also be taken as contraceptive

*GAHT* gender-affirming hormone therapy, *GnRH* gonadotropin releasing hormone

Because GAHTs are used off-label, it is crucial to inform the patient about their adverse effects, most notably their possible effects on fertility. An important aspect of patient care (frequently overlooked, particularly among transitioning adolescents) when considering GAHT is to discuss the possibility of fertility preservation prior to initiating therapy [23].

GAS encompasses a variety of procedures tailored to the needs of transgender individuals. Feminising surgeries can include facial feminisation, voice and thyroid cartilage modification, mammoplasty, vaginoplasty or coloplasty and orchiectomy. Masculinising surgeries may involve thoracic masculinisation, hysterectomy, oophorectomy, metoidioplasty and phalloplasty. It is important to take into account the change in oncological risk due to the different surgeries a patient may have undergone [24].

Since GAC results in significant changes, patients may require extensive consultations with treating physicians. However, research suggests that only a small fraction of transgender patients with cancer adequately discuss treatment plans with their physicians, such as the impact of radiotherapy on the results of previous gender-affirming breast augmentations or decisions around prophylactic mastectomies during oncological surgeries [1].

## Cancer risk in transgender people

Transgender individuals frequently encounter barriers to cancer prevention and screening (see ‘Screening and Diagnostic Challenges’ below) and face significant challenges in accessing and receiving timely, quality cancer care [20]. As such, they are considered to be at a potentially higher risk of cancer compared with the general population. Lifestyle and environmental risk factors, and risk factors associated with GAC all contribute to the oncogenic risk in transgender people.

### Lifestyle and environmental cancer risk factors

Transgender individuals have traditionally faced unique lifestyle risk factors that can significantly influence their overall cancer risk [4]. Human papillomavirus (HPV) is recognised as the most common sexually transmitted infection globally, with certain serotypes posing high carcinogenic risks. Approximately 5% of all cancers worldwide, including those of the oropharynx, anus, penis, vagina, cervix and vulva, are attributed to HPV [24]. The burden of HPV and its oncogenic potential is of particular concern within transgender communities, where prevalence rates are high, particularly in populations such as transgender sex workers or those from developing countries. A study in the USA reported an HPV prevalence rate among HPV-unvaccinated transgender women of 88.6% (from anal specimens) and 9.1% (from oral specimens) [25]. Data available from Brazil, also for HPV-unvaccinated transgender women, reported a prevalence rate of 77.9% (from anal specimens) [26]. Additionally, the intersection of HPV prevalence and HIV status further complicates the oncogenic risk, as illustrated in this study, where significantly more transgender women who were HIV-positive had high-risk HPV serotypes than those who were HIV-negative (75.4% vs 48.7%; *P* < 0.001) [26]. These figures highlight the urgent need for targeted population-based studies to better understand and address this health concern [1].

The surgical construction of a neovagina, particularly by using heterotopic penile skin in transgender women, presents specific risks for HPV exposure and subsequent cancer development [27, 28]. The penile skin, repurposed during vaginoplasty, may carry a higher susceptibility to HPV infections, potentially leading to squamous cell carcinoma. A study examining neovaginal swabs from transgender women post-vaginoplasty found that 20% of sexually active participants tested positive for high-risk HPV, underscoring the oncogenic risk associated with the surgical site [24].

The prevalence of HIV among the transgender population is disproportionately high, reflecting the significant health disparities this community faces. A systematic review of 88 studies conducted in the USA found that the laboratory-confirmed HIV infection rates among transgender individuals were 9.2%, while self-reported rates were significantly higher at 16.1% [29]. Transgender women had a much higher rate of HIV infection (14.1%) compared with transgender men (3.2%).

Substance abuse represents a significant health challenge within the transgender community, characterised by higher prevalence rates and associated risks, including cancer risk, compared with the general population. Research indicates that the prevalence of tobacco use, alcohol consumption and illicit drug use among transgender people is higher than in the general population [30, 31]. For instance, Fredriksen-Goldsen and colleagues found that 15% of transgender individuals over 50 years of age in the USA use tobacco—higher than the 9% observed in the non-transgender population [31]. Similarly, a systematic review found that rates of heavy episodic drinking (“binge drinking”) and the use of illicit substances such as cocaine and amphetamines were notably higher among transgender than cisgender populations [30]. Neither of these studies reported cancer incidence, however, it is well established that tobacco use and alcohol abuse are leading contributors to various cancers. HCPs should counsel transgender and gender-diverse individuals on the risks associated with tobacco use and to advocate for tobacco/nicotine abstinence, particularly prior to undergoing gender-affirming surgeries [21].

## Screening and diagnostic challenges

Transgender people often face significant barriers when accessing cancer screening programs (such as those for cervical and BC), resulting in lower adherence to these essential programs, and consequently, diagnosis at later stages and poorer prognoses [5, 20, 45]. Lower rates of screening for BC and cervical cancer compared with cisgender individuals [11, 47, 48] and the general population [49] have been observed. Transgender patients are also less likely to receive cancer treatment [45] and thus experience worse outcomes [45, 50]. For example, transgender patients with BC experience a threefold higher BC recurrence rate than cisgender heterosexual individuals [50]; survival rates post-diagnosis are poorer for transgender individuals, especially for cancers such as non-Hodgkin lymphoma, prostate and bladder cancers [45].

Differences in screening rates may be explained, at least in part, by the processes used in screening programs. In Italy, for instance, population-based screening invitations are often sent on the basis of biological sex at birth, leading to exclusion of transgender individuals after legal gender reassignment [20]. Although there is no reference to transgender people in the Spanish national guidelines, some regional guidelines do provide guidance for the healthcare of transgender individuals [51–53], e.g., there is a recommendation for transgender men to follow the same recommendations as cisgender women for breast and cervical cancer screening [52]. We recommend that, in an effort to improve early detection and increase screening rates among transgender populations, healthcare systems must adopt more inclusive and gender-sensitive policies and training programs that address and mitigate these disparities. This includes ensuring that screening recommendations are conducted based on the presence of specific organs rather than assumed gender norms [54]. All screening should be tailored to the individual’s specific medical history and anatomical considerations. We advise that HCPs must engage in open, informed discussions with transgender patients about the risks and benefits of cancer screenings, and consider the emotional and psychological comfort of their patients during these screenings.

Due to limited data on the health status and outcomes of transgender people with cancer, there are no standardised global guidelines on cancer screening in this patient group. Recommendations on cancer screening are available from expert opinion and reviews of the available scientific literature [10, 55–57], as well as from various academic associations (e.g., the NCCN [6], the ACR [58], and the WPATH [21]. Important considerations for screening in relation to selected cancers are described below.

### Breast cancer

BC screening recommendations are generally based on the sex assigned at birth, risk factors (e.g., family history, pathogenic genetic variants) and GAHT [6, 21, 57, 58]. Although strict adherence to screening programs is warranted, clinical advice regarding screening should be individualised for transgender individuals based on characteristics of the GAS or risk-reducing surgery.

WPATH recommend that transgender men who have not had gender-affirming chest surgery should participate in BC screening programs developed for cisgender women [21]. The ACR do not recommend breast screening for transgender men who have undergone bilateral mastectomy because, based on data from BC risk reduction in high-risk cisgender women, the risk of BC is thought to be significantly reduced [58]. However, annual chest wall examinations are recommended to monitor for any changes [8]. WPATH acknowledge that the risk of BC is not entirely eliminated in these individuals, but screening is advised only among those who have a family history of BC or who are identified as *BRCA* gene mutation carriers [21]. The recent US Preventive Services Task Force recommendations on screening do not discriminate between transgender men with or without intact breasts, but include all persons AFAB in their recommendation to undergo 2-yearly screening mammography between the ages of 40 and 74 years in individuals at average risk [57].

WPATH recommends that transgender women who have received oestrogen GAHT should be screened as per guidelines for cisgender women, taking dosage, treatment duration and age, into account [21]. ACR guidance for transgender women depends on hormone use, age and risk-level; in individuals with past or current hormone use of ≥ 5 years’ duration, mammography/digital breast tomosynthesis screening may be considered in those aged ≥ 40 years and at average risk, and is recommended for those aged 25–30 years and at high-risk [58]. High-risk factors include personal or family BC history, chest irradiation between the ages of 10–30 years, untested individuals with a first-degree relative with genetic risk and those with a genetic predisposition to BC [58].

**Fig. 1:**
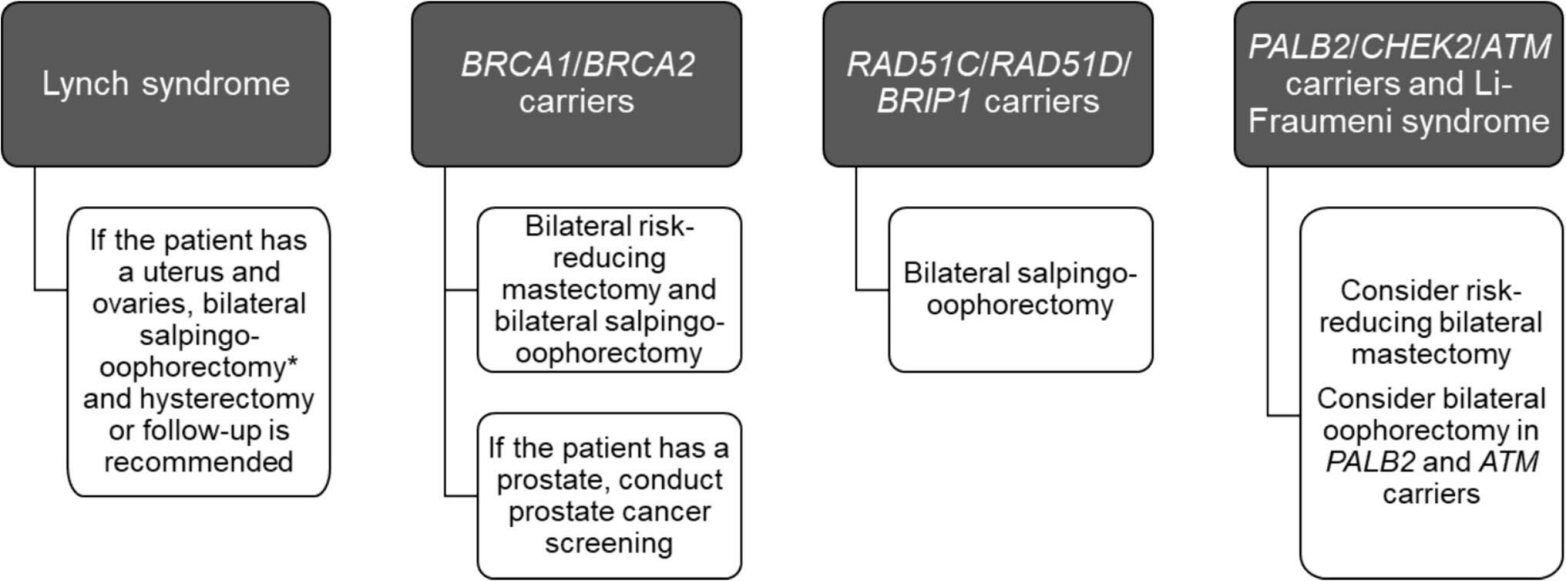
Different surgery recommendations for individuals undergoing gender-affirming surgery according to the presence of different mutations that confer cancer risk [38]. * No clear benefit of bilateral salpingoophorectomy in *PMS2* pathogenic variant carriers and lower benefit in *MSH6* pathogenic variant carriers. *ATM* ataxia-telangiectasia mutated, *BRCA* BReast CAncer gene, *BRIP1* BRCA1-interacting protein 1, *CHEK2* checkpoint kinase 2, GAS gender affirming surgery, *PALB2* Partner and localizer of BRCA2, *RAD51D* RAD51 Paralog D

In transgender individuals carrying pathogenic genetic variants predisposing to familial cancer, decisions on screening should follow clinical practice guidelines that provide specific guidance on the particular variant(s) carried (e.g., the NCCN Guidelines) [6]. In these individuals, GAS might be modified and risk-reduction surgery included after individual discussion (Fig. 1).

### Cervical cancer

The 8^th^ edition on standards of care from the WPATH recommends cervical cancer screening be provided for individuals with a cervix according to age-based regional practices and guidelines [21]. Spanish guidelines recommend regular cervical cancer screening between the ages of 25 and 65 years, either using Papanicolaou (pap) smears every 3 years or HPV testing every 5 years [52]. Importantly, testosterone therapy may influence the outcome of pap smears and HCPs should be aware of this [59, 60]. There is the potential for benign morphologic alterations to be misinterpreted as abnormalities: for example, severe atrophic changes for squamous dysplasia, transitional cell metaplasia for high-grade dysplasia in a lesion and inflammatory changes [61]. HCPs should be aware that such mis-categorisations are likely to have significant clinical implications in the transgender population [61].

If the cervix has been removed and there is no history of cervical cancer in transgender males, cervical cancer screening is unnecessary. It is recommended that transmasculine patients > 21 years undergo an annual pap smear if the cervix is present and transgender women with a neovagina do not require cervical cancer screening [10].

### Prostate cancer

It cannot be assumed that the risk of PC is entirely eliminated by anti-androgen treatment in transgender women [40]. The role of screening for PC in transgender women is not well established, and notably, evidence for prostate screening excludes data from transgender women [62]. Prostate screening is less frequently utilised by transgender women due to a combination of medical, psychological and social barriers, including insufficient awareness among both patients and HCPs about the risks and necessary preventive measures against PC in transgender women [63].

WPATH do not currently include specific recommendations regarding PC screening in transgender women [21], but others have suggested that the same screening guidelines for PC be followed for transgender women as for cisgender men [10]. Given the lower incidence of PC but worse prognosis in transgender women, they should be offered an individualised decision-making process around regular screening such as prostate-specific antigen (PSA) testing [64]. Screening guidelines advise that individuals aged 40–50 years with a family history of PC or other high-risk features should undergo annual PSA testing, those without such risk features should undergo an annual PSA test between the ages of 50 and 75 years, and screening is optional in those older than 75 years (depending on life expectancy) [10]. The PSA upper limit of normal in patients with a prostate receiving GAHT is considered to be 1 ng/mL [10]. It is also essential to discuss with transgender women the long-term benefits of PSA screening and digital rectal exams [64].

## Recommendations for improving oncological care for transgender patients

HCPs must develop a deeper understanding of the unique needs and barriers faced by this population, and act to address these needs and assist in reducing these barriers. Inequities exist not only in healthcare delivery but also in clinical research [4, 65]. An in-depth review of recommendations to improve cancer care and research for the transgender community is beyond the scope of this article but selected issues are highlighted in Table 3.

**Table 3:** Summary of key recommendations for improving cancer care in transgender individuals [20, 21, 65]

**Cancer research and data**
Increase the participation of transgender individuals in cancer clinical trials
Include gender identity measures in population-based studies
Include the routine collection of gender identity data by HCPs
Research should focus on the holistic care of patients, including their psychosocial needs
**Healthcare processes, systems and institutions**
Policies should promote inclusive cancer care, from terminology used in clinical care encounters and medical records, to the labelling and use of clinics and rooms
Screening guidelines should acknowledge and address the specific needs and risks of transgender individuals
HCPs and personnel should receive cultural awareness training in relation to transgender individuals
Cancer healthcare clinicians should receive education and training about transgender health issues
**Clinical practice**
Use an individual’s preferred names/pronouns/adjectives in clinical encounters and record keeping, in a consistent manner
Primary care practitioners should maintain up-to-date medical records relating to organs present, gonadal surgical history and hormone use
Offer procedures that avoid retraumatising individuals, where possible
Individualise treatment approaches, particularly when cancer treatment needs to be considered in the context of the needs of the patient relating to GAHT
Provide support for the individual’s psychological needs

*GAHT* gender-affirming hormone therapy, *HCPs* healthcare professionals

Transgender individuals and other gender minorities are deeply under-represented in pivotal BC treatment trials [66]. Enhancing the participation of transgender individuals in cancer clinical trials is crucial. This requires changes to clinical trial protocols, such as the use of gender-inclusive language and the avoidance of applying exclusion criteria such as HIV infection and the use of GAHT, unless these are scientifically justified [20]. Additionally, inclusion of gender identity measures in population-based surveys is needed to expand understanding of the healthcare needs and experiences of transgender individuals [67]. There remains a significant gap in the collection of sexual orientation and gender identity data, which is critical for tailoring cancer care and improving treatment outcomes [20]. As such, HCPs should be trained and supported to collect these data effectively.

The Assisi recommendations by the Italian Association of Medical Oncology highlight the urgent need for comprehensive policies that integrate a gender-sensitive approach into oncological care [20]. For example, a transgender patient’s sense of comfort and safety in clinical settings could be improved by establishing gender-neutral labelling of services or clinics and other settings, and by using preferred names/pronouns/adjectives when addressing transgender individuals and when entering data in medical records [20, 65]. Trauma-informed care approaches are essential, particularly for individuals who have experienced violence, to minimise re-traumatisation during clinical procedures [4]. Offering less invasive options, such as self-administered pap smear tests, could significantly reduce barriers for cancer screening. Additional actions are needed to make participation in clinical trials more inclusive [4].

Current cancer screening guidelines are largely based on data derived from the cisgender population, which may not be entirely applicable to transgender individuals [20]. Developing guidelines that consider the specific needs and risks associated with transgender patients is imperative. In addition, further research efforts should explore the relationship between GAHT and cancer risk, as current evidence is inconclusive, despite some studies suggesting a potential increase in risk [20]. Educating transgender individuals, especially youth, about preventive measures and modifiable cancer risk-reducing factors such as tobacco and alcohol use, and the importance of vaccinations against HPV and hepatitis B, is vital [20].

Significant gaps remain in the existing literature, particularly concerning the psychosocial aspects of cancer care for transgender patients [68]. Future research should focus on addressing these gaps to provide a holistic approach that supports the well-being of transgender individuals.

Finally, in transgender patients diagnosed with cancer (particularly BC), the coordination of GAC and cancer treatment must be considered carefully. Endocrine-based treatment of hormone-receptor positive BC in transgender patients may interfere with GAHT; however, there is a lack of clear evidence about this issue [69], and the impact of GAHT on BC prognosis is largely unknown. Ideally, cancer treatments should be individualised to meet both the medical and gender-affirming needs of the patient, and to balance the risk and benefits of combining GAHT with endocrine-based cancer treatments [69]. As such, treatment decisions should be made according to patient preference and risk of disease progression; either combining GAHT with BC endocrine treatment or interrupting GAHT to avoid the pro-tumour effects of oestrogens or testosterone. In transgender women receiving oestrogens, aromatase inhibitors would not be effective [69]; discontinuation of oestrogens should be considered in transgender women receiving cancer-directed endocrine treatment [70]. In transgender men receiving testosterone, aromatase inhibitors might block testosterone effects [69].

## Conclusions

This review highlights the significant oncological challenges and disparities faced by transgender individuals, underscoring the urgent need for tailored healthcare protocols that address their unique needs. An integrated approach to care, encompassing both GAC and vigilant cancer management, is essential.

To bridge the current gaps in knowledge and practice, concerted efforts in research, education and policy reform are necessary to ensure that transgender individuals receive equitable and effective cancer care. Future directions should focus on expanding accessible, transgender-specific health data, improving HCP education on transgender health issues and developing inclusive clinical practices that enhance patient care and outcomes.

## Data Availability

Data sharing is not applicable to this article as no datasets were generated or analysed during the current study.
